# Ferroptosis in Parkinson’s disease: a review of molecular mechanisms and emerging therapeutic strategies

**DOI:** 10.3389/fnins.2026.1780573

**Published:** 2026-02-20

**Authors:** Lingling Wang, Yue Zhang, Lei Guo

**Affiliations:** 1 Department of Neurology, Yantaishan Hospital, Binzhou Medical University, Yantai, Shandong, China; 2 Health Center, Shandong Provincial Chronic Disease Hospital, Qingdao, Shandong, China

**Keywords:** ferroptosis, iron metabolism, neurodegeneration, Parkinson’s disease (PD), therapeutic targets

## Abstract

Parkinson’s disease (PD) is a progressive neurodegenerative disorder characterized by the loss of dopaminergic (DA) neurons in the substantia nigra and the presence of Lewy bodies containing aggregated *α*-synuclein (α-syn). While these pathological hallmarks are well-established, the mechanisms underlying neuronal death remain incompletely understood. Emerging evidence highlights ferroptosis, an iron-dependent form of regulated cell death driven by lipid peroxidation, as a critical pathway in PD pathogenesis. This review synthesizes recent advances elucidating the synergistic interplay between *α*-syn aggregation and ferroptosis. We detail how α-syn aggregation not only directly induces ferroptosis but also disrupts iron homeostasis, while iron accumulation in turn accelerates *α*-syn fibrillation and oxidative stress, forming a vicious cycle that propagates neurodegeneration. Furthermore, we explore the amplifying role of glial cells—microglia and astrocytes—in this process through the promotion of neuroinflammation, oxidative damage, and dysregulation of iron metabolism. Finally, we discuss promising therapeutic strategies targeting this *α*-syn-ferroptosis axis, including α-syn aggregation inhibitors, iron chelators, and glia-modulating agents, highlighting their potential as disease-modifying interventions. Together, these insights underscore ferroptosis as a central mechanism in PD and offer new avenues for developing targeted therapies.

## Introduction

Parkinson’s disease (PD) is one of the most common neurodegenerative disorders, marked by the progressive loss of dopaminergic (DA) neurons and accompanying motor dysfunction. Despite extensive research, the underlying mechanisms of PD pathogenesis remain incompletely understood. Hallmark pathological features include degeneration of DA neurons in the substantia nigra and the presence of Lewy bodies (LBs) within surviving neurons (Sulzer et al., 2017). The nigrostriatal pathway, part of the basal ganglia motor circuit, becomes impaired as dopamine levels drop, leading to characteristic motor symptoms such as bradykinesia, rigidity, and resting tremor (Herrero et al., 2002).

Clinical manifestations of PD are highly heterogeneous (Tolosa et al., 2021), adding complexity to the development of therapeutic strategies. Current treatments primarily aim to restore dopaminergic signaling through levodopa, anticholinergic agents, and dopamine receptor agonists (Armstrong and Okun, 2020). However, these approaches offer only symptomatic relief and do not slow disease progression or provide neuroprotection. There is a pressing need to elucidate the molecular foundations of PD to facilitate the development of novel disease-modifying therapies.

Ferroptosis, an iron-dependent, regulated form of cell death driven by lipid peroxidation, has emerged as a critical mechanism in the pathogenesis of PD (Dixon et al., 2012). Notably, this pathway substantially overlaps with, and is now often considered synonymous to, a previously described oxidative stress-induced neuronal death process termed “oxytosis.” The consolidated term “*oxytosis/ferroptosis*” has been adopted in the neuroscience literature, reflecting a consensus that these represent the same fundamental cell death pathway (Lewerenz et al., 2018). This conceptual integration is especially relevant in PD, in which hallmark features of *oxytosis/ferroptosis*—including oxidative stress, iron accumulation, and glutathione depletion—are prominently observed (Lewerenz et al., 2018).

This review examines how ferroptosis serves as a core mechanism in PD, moving beyond a mere consequence to a fundamental driver of pathology. We discuss the synergistic vicious cycle between *α*-syn aggregation and iron dysregulation, which amplifies lipid peroxidation and ferroptotic cell death. Additionally, we explore the contributing roles of microglia and astrocytes in propagating neuroinflammation and disrupting iron and antioxidant balance. Finally, we evaluate promising therapeutic approaches targeting this α-syn–ferroptosis axis, including aggregation inhibitors, iron chelators, and glial modulators, assessing their potential to alter disease course and address current treatment limitations.

## 2 Materials and methods

This narrative review was undertaken by systematically searching the PubMed and Web of Science for pertinent literature published up to June 2025. The search strategy employed a combination of the following keywords and MeSH terms: “Parkinson’s disease,” “ferroptosis,”“lipid peroxidation,” “alpha-synuclein,” “microglia,” “astrocytes,” “therapeutics,” and “neuroprotection.” The inclusion criteria focused on original research articles, reviews, and clinical trials published in English that elucidated molecular mechanisms, pathological features, or therapeutic strategies related to ferroptosis in PD. Relevant references from the retrieved articles were also screened to ensure comprehensive coverage. Data extraction and synthesis were performed thematically to construct the mechanistic framework and therapeutic overview presented in this review.

## 3 The pathophysiology of Parkinson’s disease

PD is the second most common neurodegenerative disorder after Alzheimer’s disease (AD). Global data analysis shows that while its crude prevalence increased by 74% between 1990 and 2016, the age-standardized increase was only 22% (Dorsey et al., 2018). The etiology of PD is multifactorial, involving both genetic and environmental factors. Risk factors (such as pesticides) and protective factors (such as physical activity and smoking) are thought to influence the development of PD, although establishing causality remains challenging due to the extended prodromal period (Ben-Shlomo et al., 2024). Further studies are needed to refine our understanding of its epidemiology and to elucidate its role in the complex pathogenesis of PD.

With recent advancements in understanding the genetic architecture of PD, an enhanced understanding of its genetic mechanisms is anticipated. It is recognized that nearly all PD cases are likely influenced by identifiable genetic factors, with specific genetic variants varying in frequency and effect size across individual cases. To date, 100 distinct genes or loci have been strongly associated with PD susceptibility. Three autosomal dominant genes (SNCA, LRRK2, and VPS35) and three autosomal recessive genes (PRKN, PINK1, and DJ1/PARK7) have been confirmed as causes of PD, along with several other genes linked to fewer cases or families (Blauwendraat et al., 2020).

*α*-syn, a small soluble protein (140 amino acids) encoded by the SNCA gene, is widely recognized as a central component in the pathogenesis of PD. α-syn pathology is hypothesized to spread transsynaptically, at least partially, along neuroanatomical circuits (Sharma and Burré, 2023; Chahine et al., 2020). This hypothesis has been supported by studies in mice, rats, and non-human primates, demonstrating *α*-syn propagation from the periphery to the central nervous system (CNS) and vice versa, as well as from the brain to other organs (Luk et al., 2012; Recasens et al., 2018; Chu et al., 2019; Takahashi et al., 2025). Moreover, the occurrence of premotor gastrointestinal symptoms, which may precede Substantia Nigra (SN)-associated motor symptoms, further supports the concept of pathological spread (Ross et al., 2012). Evidence suggests that the accelerated accumulation of pathogenic α-syn plays a critical role in the onset and progression of PD. However, further studies are required to elucidate the precise mechanisms underlying its pathogenesis.

**Figure 1:**
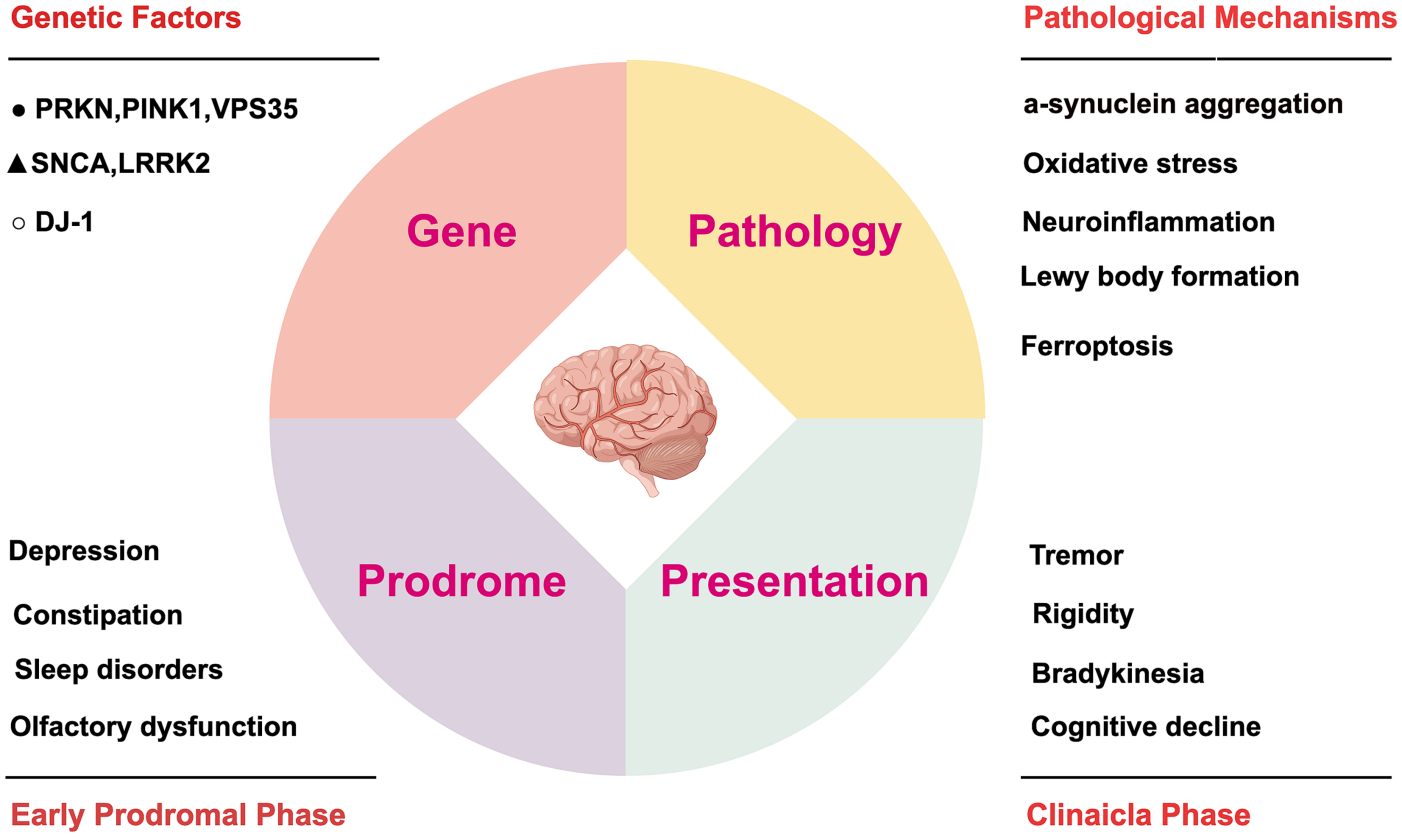
Pathogenic factors and their impact on disease progression in PD. The figure illustrates the molecular pathogenesis of PD alongside the corresponding timeline of clinical symptom progression. At the molecular level, key genetic mutations are categorized by their associated risk levels for PD pathogenesis: ▲ high-risk, ● moderate-risk, and ○ low-risk factors. The progressive development of motor and non-motor symptoms throughout the disease course is also delineated. This figure was created using Figdraw (https://www.figdraw.com). SNCA, alpha-synuclein; LRRK2, leucine-rich repeat kinase 2; VPS35, vacuolar protein sorting 35; PRKN, parkin RBR E3 ubiquitin-protein ligase; PINK1, PTEN-induced putative kinase 1; DJ-1, parkinsonism-associated deglycase.

Despite recent advances in our understanding of these pathophysiological processes, several questions remain unanswered. The pathological features of PD, particularly iron accumulation, oxidative stress, and mitochondrial dysfunction, bear striking similarities to the hallmarks of ferroptosis. Gaining a deeper understanding of the molecular mechanisms and regulatory pathways involved in ferroptosis may offer valuable insights into PD pathogenesis. In Figure 1, we have illustrated the pathogenic mechanisms of PD and summarized the high-risk factors contributing to disease progression.

## 4 Ferroptosis

Ferroptosis is an iron-dependent, regulated form of cell death driven by the lethal peroxidation of membrane lipids, distinct from apoptosis, necrosis, and autophagy. Its conceptual framework in neuroscience originated from the earlier characterization of “oxytosis” in models of glutamate toxicity (Lewerenz et al., 2018). The now-integrated term *oxytosis/ferroptosis* reflects this shared pathway (Lewerenz et al., 2018), which was formally defined in 2012 (Stockwell et al., 2017). The core machinery governing this process rests on three interconnected pillars: cellular iron metabolism, the peroxidation of membrane lipids, and endogenous antioxidant defense systems (Galy et al., 2024; Tang et al., 2021; Fang et al., 2023; Chen et al., 2023). Critically, each pillar has a direct counterpart in PD pathology: iron accumulation in the substantia nigra, elevated lipid peroxidation products, and a profound depletion of glutathione. This alignment underscores why *oxytosis/ferroptosis* is implicated as a critical executioner of dopaminergic neurons.

The molecular details of these pillars are as follows. Membrane phospholipids containing oxidizable polyunsaturated fatty acids (PUFAs) are primary targets for peroxidation, which can be initiated via enzymatic routes involving lipoxygenases or non-enzymatic mechanisms propelled by reactive oxygen species (ROS). A critical contributor is the redox-active labile iron pool (Fe^2+^), which catalyzes the Fenton reaction, converting lipid hydroperoxides into highly reactive hydroxyl radicals that propagate oxidative membrane damage. Cellular protection against ferroptosis is mediated by several parallel antioxidant systems. The canonical pathway relies on cystine uptake through system Xc^−^ for glutathione (GSH) synthesis, which serves as a cofactor for glutathione peroxidase 4 (GPX4). Independent parallel systems further enhance ferroptosis resistance. The Ferroptosis Suppressor Protein 1 (FSP1)/Coenzyme Q10 (CoQ10) axis regenerates the radical-trapping antioxidant ubiquinol within membranes, while the GTP Cyclohydrolase 1 (GCH1)/Tetrahydrobiopterin (BH4) pathway modulates lipid metabolism and CoQ10 levels to suppress peroxidation. These coordinated networks collectively maintain redox equilibrium and define cellular susceptibility to ferroptosis (Zhang Y. et al., 2025; Guo et al., 2024).

Emerging research continues to reveal novel regulatory axes and crosstalk between ferroptosis and other cell death modalities. For instance, the bacterial metabolite Pseudomonas quinolone signal (PQS) activates host histidine methyltransferase carnosine-N-methyltransferase (CNMT), leading to epigenetic upregulation of transferrin receptor 1 (TFR1) and enhanced iron uptake, thereby promoting ferroptosis (Jia et al., 2025). Additionally, ferritin-targeted degraders such as ATTEC compounds induce ferroptosis by mimicking ferritinophagy, liberating stored iron and hydrogen peroxide to fuel Fenton chemistry (Song et al., 2025).

**Figure 2:**
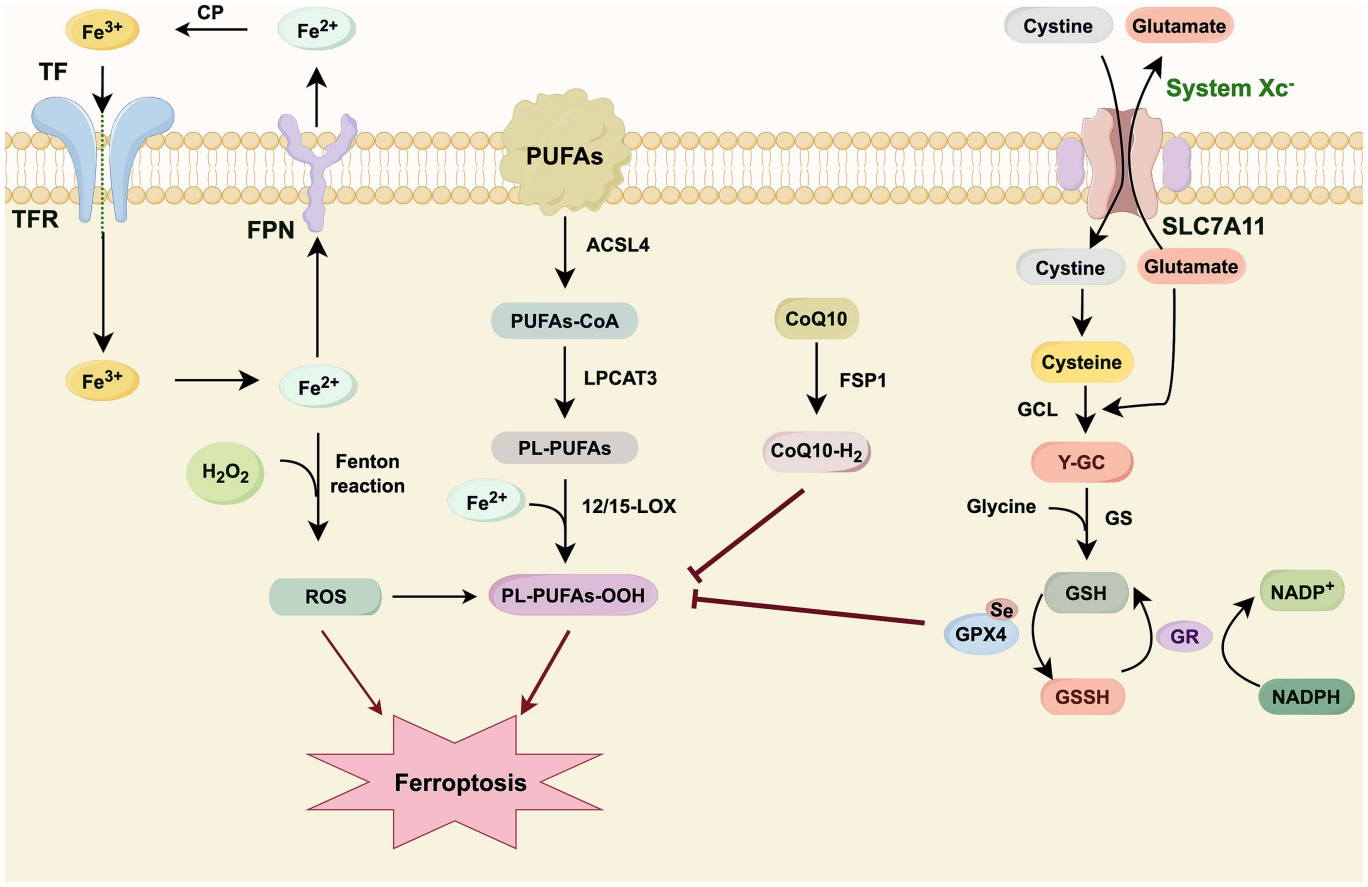
This schematic diagram delineates the core mechanistic framework of ferroptosis, encompassing the pivotal processes of iron metabolism, lipid peroxidation, and cellular antioxidant defense. Intracellular iron accumulation is initiated by transferrin (TF)-bound iron uptake, with ferroportin (FPN) facilitating iron export. Labile Fe^2+^ catalyzes the generation of ROS via the Fenton reaction with hydrogen peroxide (H_2_O_2_), thereby initiating lipid peroxidation. The peroxidation-susceptible lipid pool is shaped by ACSL4 and LPCAT3, which sequentially activate and incorporate PUFAs into membrane phospholipids (PL-PUFAs). These PL-PUFAs are subsequently oxidized by lipoxygenases (e.g., 12/15-LOX) to form lipid hydroperoxides (PL-PUFAs-OOH), the terminal executors of ferroptotic cell death. Cellular protection against ferroptosis is primarily mediated by the System Xc–GSH–GPX4 axis. The System Xc^−^ antiporter, comprising subunits SLC7A11 and SLC3A2, imports extracellular cystine in exchange for glutamate. This cystine is reduced to cysteine, a rate-limiting precursor for the synthesis of glutathione (GSH). Glutathione synthetase (GS) then catalyzes GSH formation. GPX4 utilizes GSH as a cofactor to reduce cytotoxic lipid hydroperoxides to inert alcohols, thereby safeguarding membrane integrity. The glutathione redox cycle is maintained by glutathione reductase (GR), which regenerates GSH from its oxidized form (GSSG) using NADPH. In parallel, a GPX4-independent pathway orchestrated by FSP1 suppresses ferroptosis. FSP1 catalyzes the regeneration of ubiquinol (CoQ_10_-H_2_) from ubiquinone (CoQ_10_), which acts as a lipophilic radical-trapping antioxidant within the plasma membrane to halt the propagation of lipid peroxidation. This figure was created using Figdraw (https://www.figdraw.com). TF, transferrin; TfR, transferrin receptor; Fe^3+^, ferric iron; Fe^2+^, ferrous iron; FPN, ferroportin; PUFAs: polyunsaturated fatty acids; ACSL4: acyl-CoA synthetase long-chain family member 4; LPCAT3, lysophosphatidylcholine acyltransferase 3; PL-PUFAs: phospholipid-polyunsaturated fatty acids; 12/15-LOX, 12/15-lipoxygenase; ROS, reactive oxygen species; CoQ_10_, coenzyme Q_10_; FSP1, ferroptosis suppressor protein-1; CoQ_10_-H_2_, reduced coenzyme Q_10_; SLC7A11, solute carrier family 7 member 11; GCL, *γ*-glutamyl cysteine ligase; Y-GC, gamma-glutamylcysteine synthetase; GS, glutathione synthetase; GPX4, glutathione peroxidase 4; GSSG, glutathione; GR, glutathione reductase; GSH, glutathione; NADPH, nicotinamide adenine dinucleotide phosphate; Se, selenium.

Notably, ferroptosis exhibits significant synergy with cuproptosis and disulfidptosis, particularly through shared metabolic nodes such as GSH depletion and mitochondrial dysfunction. Integrated therapeutic strategies that co-activate these pathways, such as dual-metallic nanosystems or glucose metabolism-targeting agents, demonstrate enhanced antitumor efficacy by overcoming compensatory resistance mechanisms. This evolving understanding of ferroptosis interconnectivity underscores its role within a broader cell death network, revealing new avenues for combination therapy (Zhang Y. et al., 2025; Zhang et al., 2025a; Zhang et al., 2025b). This principle of targeting shared metabolic vulnerabilities may inform the development of multi-target therapeutic strategies for complex neurodegenerative diseases like PD. Given the multitude of molecules implicated in ferroptosis regulation, we summarize the principal regulators of cellular iron homeostasis and ferroptosis in Table 1, and illustrate the key antioxidant defense pathways relevant to PD in Figure 2.

**Table 1:** Key molecular regulators and pathways in ferroptosis.

Functional pathway	Key regulators	Core functions in ferroptosis	References
Iron metabolism and homeostasis	NCOA4	Mediates ferritinophagy, releasing free iron	Wu et al. (2023)
	IRP1/IRP2	Post-transcriptionally regulate transferrin receptor and ferritin to control iron uptake and storage	Terzi et al. (2021)
	SLC40A1	Sole known cellular iron exporter; prevents iron overload	Zhang Y. et al. (2024)
	HMOX1	Degrades heme to release free iron, potentiating ferroptosis.	Zheng et al. (2023)
Lipid peroxidation machinery	ACSL4	modulating the oxidative reactions	Ding K. et al. (2023)
	LPCAT3	Incorporates PUFAs into membrane phospholipids	Liu J. et al. (2022)
	ALOX15	Directly peroxidizes membrane phospholipids	Cai et al. (2023)
	POR	Generates ROS via NADPH, driving lipid peroxidation.	Yan et al. (2021)
Antioxidant defense systems	GPX4	Primary enzyme that reduces lipid hydroperoxides; essential ferroptosis inhibitor.	Zhang W. et al. (2024)
	SLC7A11	Imports cystine for GSH synthesis; its inhibition induces ferroptosis.	Koppula et al. (2021)
	FSP1	Generates CoQH₂, a lipophilic antioxidant that suppresses ferroptosis independently of GPX4	Bersuker et al. (2019)
	GCH1	Synthesizes BH4 and CoQ10, protecting against lipid peroxidation	Xiao et al. (2023)
	DHODH	Mitochondrial enzyme that generates CoQH₂ as a backup system for GPX4.	Mao et al. (2021)
Transcriptional and metabolic regulation	NRF2	Master regulator of antioxidant response; upregulates SLC7A11, ferritin, and other protective genes.	Dodson et al. (2019)
	p53	Dual role: can promote ferroptosis by suppressing SLC7A11 or inhibit it via other targets.	Kang et al. (2019)
	SAT1	A p53 target that upregulates ALOX15, promoting lipid peroxidation.	Dang et al. (2022)
	HSPB1	Inhibits ferroptosis by reducing iron uptake and lipid ROS production	Liang et al. (2023)

ACSL4, Acyl-CoA Synthetase Long Chain Family Member 4; ALOX15, Arachidonate 15-Lipoxygenase; BH4, Tetrahydrobiopterin; CoQH₂, Ubiquinol; DHODH, Dihydroorotate Dehydrogenase; FSP1, Ferroptosis Suppressor Protein 1; GPX4, Glutathione Peroxidase 4; GCH1, GTP Cyclohydrolase 1; GSH, Glutathione; HMOX1, Heme Oxygenase 1; HSPB1, Heat Shock Protein Beta-1; IRP1/IRP2, Iron Regulatory Protein 1/Iron Regulatory Protein 2; LPCAT3, Lysophosphatidylcholine Acyltransferase 3; NCOA4, Nuclear Receptor Coactivator 4; NRF2, Nuclear Factor Erythroid 2-Related Factor 2; POR, NADPH-Cytochrome P450 Reductase; p53, Tumor Protein p53; PUFAs, Polyunsaturated Fatty Acids; ROS, Reactive Oxygen Species; SAT1, Spermidine/Spermine N1-Acetyltransferase 1; SLC7A11, Solute Carrier Family 7 Member 11; SLC40A1, Solute Carrier Family 40 Member 1.

Although the definitive physiological functions of ferroptosis remain to be fully elucidated, growing evidence implicates it in various biological processes—including antiviral immunity, tumor suppression, aging, and development. Beyond its roles in normal physiology, ferroptosis has also been linked to the pathogenesis of multiple disorders, such as iron overload, brain trauma, organ damage, retinal degeneration, and neurodegenerative diseases (Stockwell, 2022).

Notably, ferroptosis has garnered increasing attention in the context of PD, where its core mechanisms, iron accumulation and lipid peroxidation, intersect strongly with established aspects of PD neuropathology. The selective vulnerability of DA neurons in the substantia nigra to ferroptotic damage may be attributed to their high basal oxidative stress, iron-rich environment, and particular lipid composition.

Overall, identifying novel regulators and protective mechanisms beyond previously described pathways will deepen our understanding of disease etiology. With the core ferroptosis pathway now well-established, we are poised to explore its specific contributions to PD pathogenesis. The following section summarizes key evidence connecting ferroptosis to DA neuron loss in PD, highlighting both mechanistic insights and emerging therapeutic opportunities.

## 5 Ferroptosis in PD

The historical and mechanistic underpinnings of ferroptosis in neurodegenerative diseases, including PD, trace back to pioneering studies of oxidative glutamate toxicity in neuronal cells. More than three decades ago, Maher, Schubert and colleagues delineated a non-excitotoxic, regulated cell death pathway triggered by glutamate, later termed *oxytosis*, which is characterized by GSH depletion, ROS accumulation, and calcium influx (Tan et al., 2001). Strikingly, the core features of *oxytosis*, including inhibition of the cystine/glutamate antiporter (system xc^−^), GSH loss, iron-dependent lipid peroxidation, and mitochondrial dysfunction, align precisely with the hallmarks of ferroptosis defined over a decade later (Dixon et al., 2012). It is now recognized that *oxytosis* and ferroptosis represent the same regulated cell-death pathway, with *oxytosis* describing its initial discovery in neurons and ferroptosis its subsequent characterization in non-neuronal systems (Maher et al., 2020).

Critically, the pathophysiological cascade of ferroptosis is recapitulated in multiple neurodegenerative conditions. In PD, post-mortem studies consistently reveal a selective decrease in GSH levels in the substantia nigra, a change that precedes overt neurodegeneration and correlates with disease severity. Elevated lipid peroxidation products and increased lipoxygenase (LOX) activity have also been documented in PD brains, further implicating the ferroptosis axis in DA neuron loss (Maher et al., 2020). These overlaps strongly support the view that ferroptosis is not merely a cell-culture phenomenon but a relevant contributor to *in vivo* neurotoxicity in aging and disease.

Translating this mechanistic understanding to PD pathogenesis, emerging evidence underscores the central role of ferroptosis. The progressive degeneration of DA neurons in the substantia nigra is accompanied by elevated iron levels and diminished glutathione content, both of which heighten cellular susceptibility to ferroptosis. Notably, iron accumulation can be detected in presymptomatic PD stages via magnetic resonance imaging, and pilot clinical studies suggest that iron chelation may confer therapeutic benefit. The intrinsic link between dopamine metabolism and ROS generation further reinforces the relevance of oxidative stress in PD. Experimentally, GPX4-knockout mice demonstrate that inactivation of this key ferroptosis-regulating enzyme triggers neuronal loss accompanied by lipid peroxidation—a phenotype attenuated by ferroptosis inhibitors such as liproxstatin-1. Moreover, human midbrain neurons show heightened sensitivity to erastin-induced ferroptosis compared with other death stimuli, and the iron chelator deferiprone mitigates dopaminergic degeneration in animal models while improving motor function in PD patients. Collectively, these findings position ferroptosis as a critical pathway in PD neurodegeneration, offering a promising target for disease-modifying interventions (Lewerenz et al., 2018).

Building on this established framework, the following sections will dissect the cell-type-specific contributions of neurons and glia to ferroptotic vulnerability and propagation in the PD brain.

### 5.1 Neuronal death: the core pathological phenotype

The pathological definition of PD, as established by the International Parkinson and Movement Disorders Society (IPMDS) Task Force, centers on the core clinical motor syndrome of parkinsonism, accompanied by the degeneration of DA neurons and the formation of LBs characterized by *α*-syn deposition (Berg et al., 2014). Misfolded and aggregated *α*-syn is the primary component of LBs. The Braak hypothesis proposes that PD exhibits a multifocal and multitransmitter-driven pathology, providing a framework for understanding its neuropathological progression (Braak et al., 2003). Indeed, *α*-syn pathology is observed not only within the brain’s dopaminergic and non-dopaminergic systems but also extensively throughout the peripheral and autonomic nervous systems. Abnormal α-syn deposition associated with LB disease has also been identified in peripheral regions, including the heart, gastrointestinal tract, submandibular glands, and skin (Beach et al., 2013; Shannon et al., 2012).

Under physiological conditions, *α*-syn exists in a soluble, non-toxic form. However, alterations in its spatial conformation can lead to the aggregation of *α*-syn, which is associated with neurotoxicity (Chen et al., 2025). The pathogenesis associated with *α*-syn aggregation depends on three distinct domains of the protein: the N-terminus (including the P1–P2 segments, which play a key role in the oligomer-to-fibril transition and harbor familial PD mutations), the hydrophobic NAC domain, and the acidic C-terminus. Notably, targeting the structurally accessible P1–P2 regions within pathogenic *α*-syn oligomers and fibrils offers a promising therapeutic strategy for selectively inhibiting aggregation, thereby supporting the development of more effective disease-modifying treatments (Bárcenas et al., 2026). Recent cryo-electron microscopy studies have further revealed a conserved *α*-syn filament architecture shared among PD, Parkinson’s disease dementia (PDD), and dementia with Lewy bodies (DLB), which is distinct from the filament structures observed in multiple system atrophy (MSA) (Schweighauser et al., 2020).

A critical relationship exists between *α*-syn aggregation and ferroptosis. Firstly, α-syn aggregation directly drives ferroptosis in PD models. One study established a cortical synucleinopathy model by overexpressing human *α*-syn in the mouse primary motor cortex (M1). Results demonstrated that α-syn aggregation activated ferroptosis, leading to the degeneration of parvalbumin-positive interneurons (PV-INs). The consequent loss of PV-INs reduced inhibitory inputs onto pyramidal neurons (PNs), disrupting cortical excitatory/inhibitory (E/I) balance and causing PN hyperexcitability and cortico-striatal pathway dysfunction. This cascade resulted in significant deficits in complex motor skill learning in the mice (Zhang B. et al., 2024). Furthermore, research reveals that *α*-syn determines DA neuron sensitivity to ferroptosis by modulating ether-phospholipid (ether-PL) membrane composition. Reducing α-syn expression decreases ether-PL levels and enhances ferroptosis resistance, while α-syn overexpression increases lipid peroxidation and ferroptosis vulnerability. These findings establish α-syn as a key regulator of neuronal ferroptosis and highlight ether-PLs as potential therapeutic targets in synucleinopathies (Mahoney-Sanchez et al., 2022). Collectively, these studies underscore the pivotal role of *α*-syn in PD-related ferroptosis and neurodegeneration, providing a rationale for therapeutic strategies targeting ferroptosis.

A complex bidirectional interaction exists between iron accumulation and α-syn pathology in PD. Iron directly binds α-syn, inducing conformational changes that promote fibrillation. Iron also regulates α-syn synthesis via the iron-responsive element (IRE)/IRP system. Iron-induced oxidative and nitrative stress triggers post-translational modifications of α-syn that enhance its aggregation. Furthermore, iron impairs *α*-syn degradation by disrupting both the ubiquitin-proteasome system (UPS) and autophagy-lysosome pathways (ALP). Conversely, α-syn dysregulates iron homeostasis: it exhibits ferrireductase activity, reducing Fe^3+^ to Fe^2+^ and elevating labile iron levels, while its overexpression redistributes iron to perinuclear inclusions. Additionally, α-syn modulates transferrin (Tf)-dependent iron uptake by regulating Tf receptor endocytosis; post-translational modifications of α-syn alter its membrane binding and vesicle trafficking, further perturbing iron flux. These multifaceted interactions form a vicious cycle that exacerbates DA neuron vulnerability (Chen et al., 2019). Subsequent research in PD mouse models demonstrated a toxic synergy between mutant *α*-syn (α-syn-A53T) and iron deposition, inducing cellular senescence in the SN prior to DA neuron loss and the onset of motor deficits (Shen Q. et al., 2024).

Iron dyshomeostasis is a central driver of neurotoxicity in PD. Evidence from a study utilizing an iron-overloaded mouse model demonstrates that iron accumulation in the midbrain induces lipid metabolism dysregulation and ferroptosis, leading to neurodegeneration. The model showed midbrain cholesterol accumulation, enhanced triacylglycerol (TAG) hydrolysis, accompanied by DA neuron loss, astrogliosis, increased *α*-syn expression, and significant elevation of lipid peroxidation and ferroptosis markers, correlating with motor deficits (Maniscalchi et al., 2024).

Clinical evidence, supported by advanced imaging techniques such as susceptibility-weighted imaging (SWI) and quantitative susceptibility mapping (QSM), confirms increased iron deposition specifically within the SN of PD patients, particularly in its ventral region, correlating with symptom severity (Martin-Bastida et al., 2017; Barbosa et al., 2015; Bergsland et al., 2019). The promise of targeting this pathway is underscored by findings that iron chelation therapies can lower nigral iron levels and provide neuroprotective effects (Jiang et al., 2017; Bar-Am et al., 2015).

Notably, the synergy extends to α-syn and lipid peroxidation. Elevated α-syn expression in neuronal precursor cells increases vulnerability to lipid peroxidation and ferroptosis by regulating ether-phospholipid membrane composition (Mahoney-Sanchez et al., 2022). Lipid peroxidation generates reactive aldehydes, such as malondialdehyde (MDA) and 4-hydroxynonenal (4-HNE), which are elevated during ferroptosis (Barayeu et al., 2023). Increased MDA levels and reduced PUFAs content have been observed in the SN of PD patients (Dexter et al., 1986). Additionally, HNE-modified proteins are enriched within LBs, and HNE itself has been detected in the cerebrospinal fluid (CSF) of PD patients (Castellani et al., 2002; Fabelo et al., 2011; Selley, 1998). Critically, these lipid peroxidation products, particularly 4-HNE and 4-oxo-2-nonenal (ONE), enhance the stability and yield of *α*-syn oligomers, promoting their pathological deposition. ONE is notably more effective than 4-HNE in promoting α-syn oligomer formation (Andersen et al., 2021). This establishes a self-reinforcing vicious cycle: α-syn oligomers instigate iron-dependent lipid peroxidation and calcium dysregulation, driving ferroptosis; in turn, the resultant oxidative stress accelerates the formation of toxic α-syn oligomers (Du et al., 2020). Collectively, these findings underscore a pathogenic synergy wherein α-syn aggregation and lipid peroxidation co-amplify each other, culminating in ferroptotic cell death and neurotoxicity.

The specific deposition of iron within the SN in PD is intrinsically linked to disrupted iron homeostasis mechanisms. The transferrin/transferrin receptor 2 (Tf/TfR2) system mediates iron transport to mitochondria in DA neurons. Mutations in the TF gene increase iron absorption and neurotoxicity, whereas deletion of TfR2 has been shown to protect against dopaminergic neurodegeneration (Mastroberardino et al., 2009; Milanese et al., 2021). Conversely, efficient iron export is crucial for maintaining neuronal iron balance. Ceruloplasmin (CP) functions in concert with ferroportin (FPN1) to export ferrous iron (Fe^2+^) while oxidizing it to the less reactive ferric form (Fe^3+^). Reduced CP activity results in iron deposition within the SN and heightened oxidative stress (Hochstrasser et al., 2005; Jin et al., 2011; Ayton et al., 2013). In essence, PD pathogenesis involves a combination of excessive iron influx and impaired iron efflux, culminating in net iron accumulation within vulnerable Substantia Nigra pars compacta (SNc) neurons.

**Figure 3:**
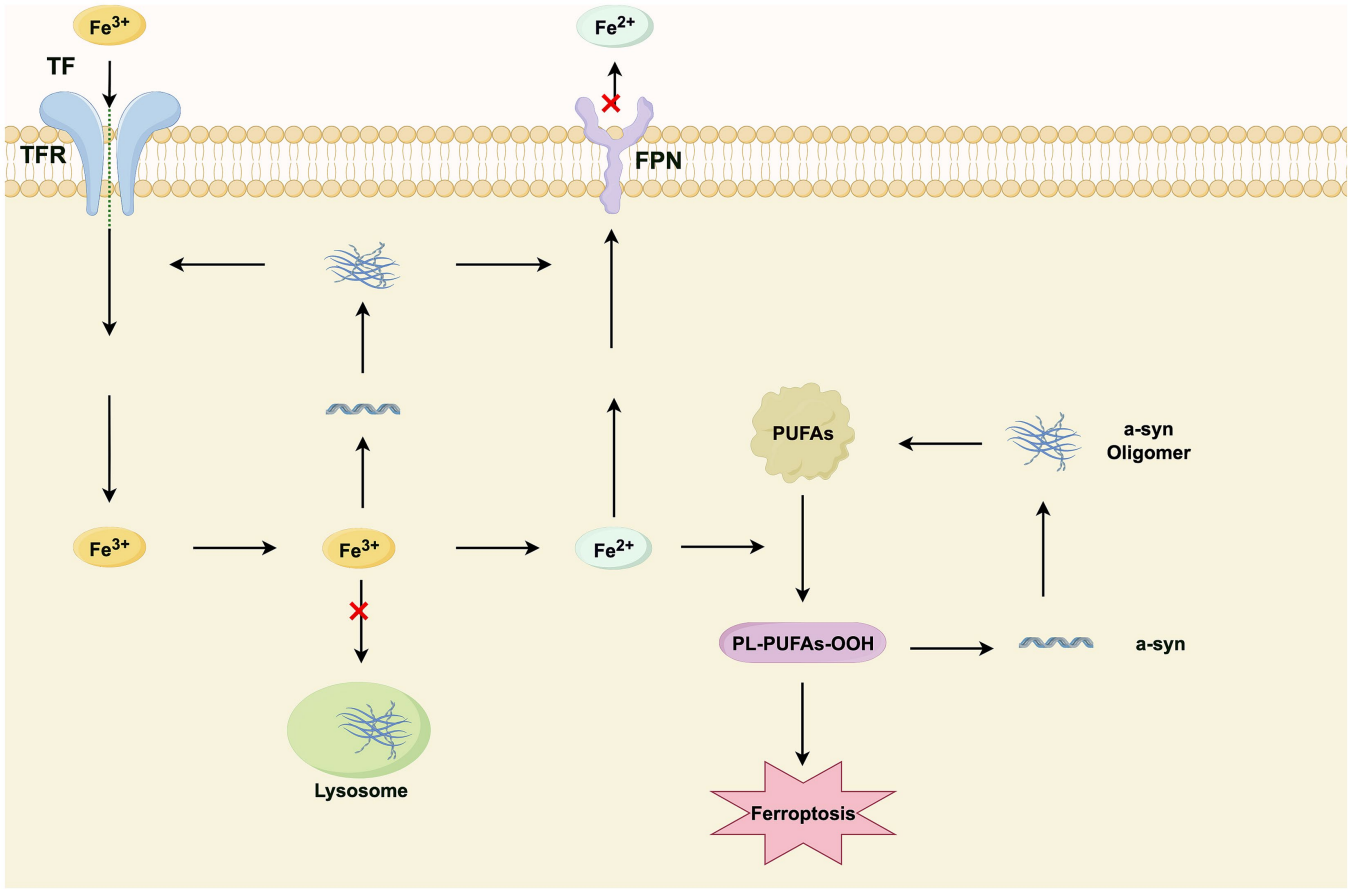
A vicious cycle of iron and α-synuclein (α-syn) in ferroptosis during PD. The schematic depicts the synergistic interplay between iron dyshomeostasis and α-syn pathology that exacerbates dopaminergic neuronal loss. The cycle is initiated by cerebral iron overload (1), which promotes α-syn aggregation and, conversely, is amplified by α-syn pathology (2). Together, they induce profound lipid peroxidation (3), the products of which further stabilize α-syn oligomers (4), thereby reinforcing the cycle. The unchecked propagation of this loop ultimately triggers ferroptosis and neuronal death.

The pathogenesis of PD is fundamentally linked to the aggregation of *α*-syn and dysregulated iron homeostasis, which synergistically drive ferroptosis and neurodegeneration. α-syn promotes lipid peroxidation and disrupts neuronal iron balance, while iron accumulation accelerates α-syn fibrillation and oxidative stress. This vicious cycle leads to DAneuron loss, particularly in the SN, and is further exacerbated by impaired iron export mechanisms (Figure 3). Therapeutic strategies simultaneously targeting α-syn aggregation and ferroptosis pathways hold promise for modifying disease progression.

### 5.2 Glial activation: amplifier of neurodegeneration

**Figure 4:**
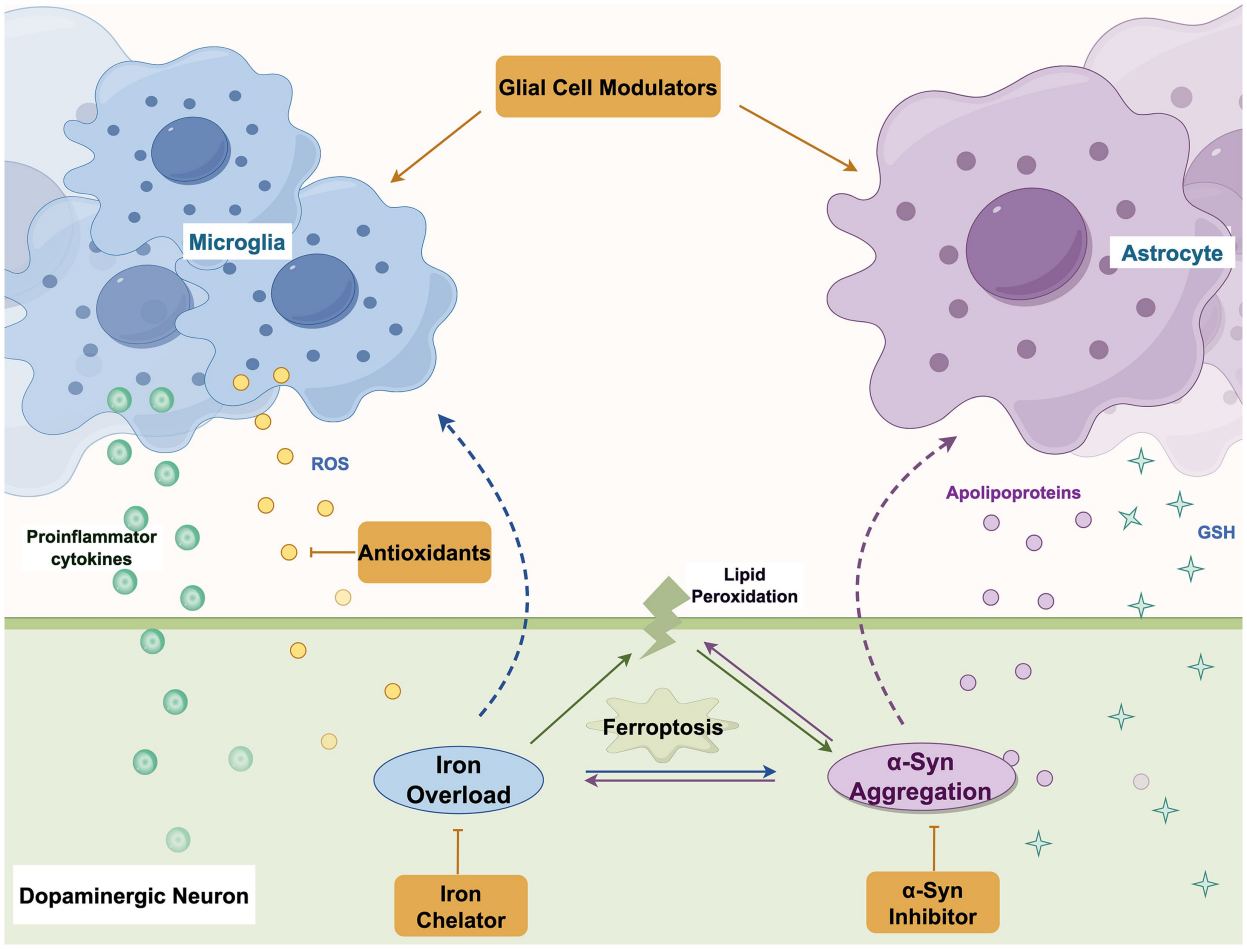
Interplay between ferroptosis and glial cells in PD. This schematic illustrates the complex interactions among neurons, microglia, and astrocytes in the context of ferroptosis during PD progression. Key processes include: (1) Neuronal release of pathological α-syn; (2) microglial activation and polarization toward a pro-inflammatory (M1) phenotype, leading to cytokine release, reactive oxygen species (ROS) production, and impaired α-syn clearance; (3) astrocytic responses, including transition to a reactive state (A1), dysregulation of antioxidant systems (e.g., GSH, GPX4), and altered neurotrophic support; (4) bidirectional crosstalk among glial cells that exacerbates neuroinflammation and oxidative stress; and (5) consequent amplification of neuronal ferroptosis via iron accumulation, lipid peroxidation, and mitochondrial dysfunction. This integrated view underscores glial cells as central amplifiers of the ferroptotic cascade and highlights potential therapeutic nodes for intervention. TF, transferrin; TfR, transferrin receptor; Fe^3+^, ferric iron; Fe^2+^, ferrous iron; FPN, ferroportin; PUFAs: polyunsaturated fatty acids; PL-PUFAs: phospholipid-polyunsaturated fatty acids; ROS, reactive oxygen species; GPX4, glutathione peroxidase 4; GSH, glutathione; a-syn, α-synuclein.

Glial cells, including astrocytes, microglia, and oligodendrocytes, represent the principal non-neuronal population in the central nervous system and critically amplify neurodegenerative processes in PD, notably through their engagement in ferroptosis pathways. As summarized in Figure 4, these cells interact dynamically within a pathogenic network that links ferroptosis, neuroinflammation, and neuronal vulnerability during PD progression. The following sections delineate the specific mechanisms through which microglia and astrocytes contribute to this self-reinforcing cycle of degeneration.

#### 5.2.1 Microglia-mediated ferroptosis propagation

Microglial iron accumulation serves as a key trigger for ferroptosis, which in turn amplifies neuroinflammation through the release of pro-inflammatory cytokines (Liu S. et al., 2022). Notably, among central nervous system cells, microglia demonstrate a particularly high susceptibility to ferroptosis compared to neurons and astrocytes. This vulnerability may compromise their ability to clear pathological α-syn aggregates via phagocytosis. Supporting this, removal of microglia from tri-culture systems significantly delayed the rate and extent of iron-induced neuronal death, underscoring the essential role of microglia in mediating ferroptotic neurotoxicity (Ryan et al., 2023). Further evidence from non-human primate models shows that intranasal administration of α-syn preformed fibrils leads to iron deposition specifically within microglia, rather than DA neurons, in the substantia nigra. This suggests that microglial iron accumulation may represent an early event triggering neuroinflammation prior to neuronal degeneration in PD (Guo et al., 2021). Thus, microglia are not only highly vulnerable to ferroptosis but also act as crucial mediators of iron-dependent neurotoxicity in PD.

Changes in the central nervous microenvironment significantly influence microglial polarization. Classically activated M1 microglia, induced by stimuli such as lipopolysaccharide (LPS) and interferon-gamma (IFN-*γ*), release neurotoxic cytokines including IL-1*β* and tumor necrosis factor alpha (TNF-*α*). In contrast, alternatively activated M2 microglia, polarized by IL-4 or IL-13, secrete protective factors like TGF-β and IL-10 that promote tissue repair and homeostasis. The dynamic balance between these phenotypes is essential for regulating neuroinflammation (Zhao et al., 2026). Importantly, iron accumulation within microglia drives them toward a pro-inflammatory M1 state, which not only exacerbates neuroinflammation and DA neuronal loss but also establishes a vicious cycle involving inflammasome activation and ferroptosis (Yu et al., 2023). Therapeutic modulation of this balance is emerging: for example, overexpression of Angiotensin-Converting Enzyme-2 (ACE2) shifts microglia toward the protective M2 phenotype, reducing dopaminergic neurodegeneration. Mechanistically, ACE2 upregulates osteoprotegerin (OPG), thereby inhibiting the non-canonical NF-κB pathway mediated by RANKL (Xu Y. et al., 2025). These findings highlight the critical role of iron in driving microglial polarization toward a pro-inflammatory state and identify phenotype switching as a viable therapeutic target.

Novel molecular regulators of microglial ferroptosis are continually being identified. A genome-wide CRISPR screen revealed that SEC24B, a gene involved in vesicle trafficking, plays a critical role in regulating ferroptosis in microglia. Its knockout markedly reduced lipid peroxidation and suppressed ferroptosis (Ryan et al., 2023). Additionally, studies in rotenone-induced PD models demonstrated that microglial complement receptor 3 (CR3) activates NADPH oxidase 2 (NOX2), leading to ROS production. This microglia-derived ROS induces neuronal iron deposition and lipid peroxidation, ultimately promoting DA neuron ferroptosis (Wang et al., 2024). Subsequent research confirmed that gp91phox, a key subunit of NOX2, promotes microglial activation, neuroinflammation, and ferroptosis, contributing to cognitive deficits in PD mice—suggesting that targeting gp91phox may offer a promising therapeutic strategy (Tian et al., 2024). These discoveries emphasize the importance of intracellular trafficking and ROS generation in microglial ferroptosis and neuroinflammation.

Mutations in Leucine-rich repeat kinase 2 (LRRK2), one of the most common genetic causes of familial PD, impair lysosomal degradation within microglia (Zhao et al., 2026). These mutations disrupt iron homeostasis by phosphorylating Rab8a, leading to the sequestration of Tf within lysosomes and potentially promoting iron accumulation and ferroptosis. Stimulation with *α*-syn upregulates LRRK2, which suppresses the protective p62-Keap1-Nrf2 pathway. This suppression results in microglial ferroptosis, release of pro-inflammatory cytokines, and subsequent DA neuron loss. Inhibiting LRRK2 activated this pathway, attenuated ferroptosis, reduced neuroinflammation, and improved motor deficits (Liu et al., 2025). Thus, LRRK2 promotes microglial activation by modulating ferroptosis and NF-κB signaling. The System Xc^−^–GSH–GPX4 antioxidant axis plays a central role in mediating LRRK2-dependent microglial ferroptosis and inflammation (Zheng et al., 2024). Overall, these studies establish LRRK2 as a key regulator of microglial iron metabolism and inflammatory responses through ferroptosis-dependent mechanisms.

DJ-1 acts as an endogenous inhibitor of ferroptosis, in part by maintaining cysteine synthesis via the transsulfuration pathway (Cao et al., 2020). Specifically, DJ-1 preserves the activity of S-adenosylhomocysteine hydrolase (SAHH), the key enzyme that hydrolyzes S-adenosylhomocysteine (SAH) to homocysteine within this metabolic cascade. Mechanistically, DJ-1 prevents the enhanced inhibitory interaction between SAHH and its negative regulator S-adenosylhomocysteine hydrolase-like 1 protein (AHCYL1), thereby promoting the formation of catalytically active SAHH tetramers. This ensures sustained homocysteine flux, which is critically converted to cysteine for GSH biosynthesis when the canonical cystine/glutamate antiporter (system xc-) is inhibited, a common trigger for ferroptosis (Cao et al., 2020). By safeguarding this metabolic bypass, DJ-1 directly supports the cellular antioxidant capacity to counteract lethal lipid peroxidation, a defining hallmark of ferroptosis.

Although the functions of DJ-1 are diverse, its role in microglia remains relatively underexplored (Dolgacheva et al., 2019). DJ-1 deficiency increases the sensitivity of microglia to dopamine, elevating monoamine oxidase (MAO) activity while reducing TREM2 expression. This trigger increased ROS production and pro-inflammatory cytokine release, ultimately exacerbating neurotoxicity (Trudler et al., 2014). Moreover, loss of DJ-1 impairs microglial function through heightened inflammatory response to α-syn, reduced α-syn uptake due to lipid raft disruption, and defective autophagy-lysosomal degradation (Nash et al., 2017). These dysfunctions collectively suggest that DJ-1 deficiency in microglia contributes to PD pathogenesis not only through enhanced neuroinflammation and oxidative stress but also potentially by compromising the intrinsic ferroptosis defense mechanism. The loss of DJ-1-mediated maintenance of the transsulfuration pathway could render microglia more vulnerable to ferroptotic damage, thereby linking its classical cytoprotective role in redox homeostasis directly to the emerging pathway of ferroptosis in neurodegeneration.

In summary, microglia play a central role in PD progression by linking iron dyshomeostasis, neuroinflammation, and ferroptosis. Key mechanisms include their high susceptibility to iron-dependent cell death, polarization toward a pro-inflammatory state under iron overload, and genetic regulation by factors such as LRRK2 and DJ-1. Targeting microglial ferroptosis and phenotype switching represents a promising therapeutic strategy to disrupt neuroinflammatory cycles and mitigate neurodegeneration in PD.

#### 5.2.2 Astrocytes: dynamic regulators of neuronal vulnerability

Astrocytes play a vital role in supporting and protecting the healthy nervous system through the release of neurotrophic factors, production of antioxidants, and clearance of neuronal waste. Under pathological conditions such as PD, astrocyte function becomes profoundly altered, significantly influencing neuronal susceptibility to ferroptosis.

Astrocytes contribute to neuronal ferroptosis through a disrupted fibroblast growth factor (FGF) signaling pathway with oligodendrocytes. Impaired FGF1/FGF9-FGFR interactions, heightened mitochondrial oxidative phosphorylation, and Ca^2+^ influx lead to excessive ROS production, ultimately promoting iron accumulation and DA neuron death in the SN (Zhang S. et al., 2025). The concept of neuroprotective (A2-type) and neurotoxic (A1-type) reactive astrocytes reflects dynamic transitional states within a continuum of the same astrocyte lineage, rather than fixed subtypes. Resting astrocytes exposed to inflammatory stimuli initially adopt a transient neuroprotective state before transitioning into a sustained neurotoxic state. This phenotypic shift is primarily regulated by the mTOR signaling pathway. In PD mouse models, reducing mTOR activity or expression levels in astrocytes diminishes their neurotoxicity and alleviates neurodegenerative phenotypes. Importantly, mTOR inhibition (e.g., via rapamycin) helps maintain astrocytes in a protective state and reduces neuronal lipid peroxidation damage (Zhang et al., 2025c). This understanding of the continuous evolution of astrocyte states offers time-sensitive therapeutic opportunities for neurodegenerative diseases. Additionally, recent research shows that cystathionine *γ*-lyase (CSE) drives astrocytes toward a neurotoxic phenotype in PD by forming a complex with Yes-associated protein (YAP), which activates Forkhead box D3(FOXD3)-mediated transcription of neuroinflammation-related genes (Zhu et al., 2024).

Reactive astrocytes counteract oxidative stress by activating nuclear factor erythroid-2-related factor 2 (Nrf2) and releasing various antioxidant molecules, such asGSH and metallothioneins (MTs). Nrf2 regulates the expression of antioxidant enzymes by binding to antioxidant response elements (AREs) and is essential for maintaining GSH homeostasis (Ovesen et al., 2014). Crucially, astrocytes employ the Nrf2/ARE pathway to suppress ferroptosis and protect DA neurons in PD models (Chen et al., 2009). Gene therapy strategies that activate the Nrf2/ARE pathway in astrocytes have been shown to reduce oxidative stress and provide neuroprotection to DA neurons in both *in vitro* and *in vivo* settings (Williamson et al., 2012). However, this protective mechanism can be impaired; disrupted crosstalk between oligodendrocytes and astrocytes, mediated by the FGF signaling pathway, weakens astrocytic defense capacity. GSH, the most abundant antioxidant in the CNS, is synthesized in astrocytes following cystine uptake (Asanuma and Miyazaki, 2021). In addition to GSH, astrocytes produce MTs, potent antioxidants that are overexpressed in the PD brain (Michael et al., 2011). Furthermore, reduced expression of melatonin receptor 1A (MT1) in astrocytes leads to increased pathological Ca^2+^ influx, exacerbating oxidative stress and suppressing the NRF2/SLC7A11/GPX4 antioxidant axis, ultimately promoting neuronal ferroptosis (Zhang S. et al., 2025).

Astrocytes are the primary producers of apolipoproteins (ApoA1, D, E, J) in the CNS, which help maintain neuronal homeostasis through lipid transport, antioxidant activity, and anti-apoptotic functions. ApoA1 promotes reverse cholesterol transport via the ABCA1 transporter, although its overexpression may paradoxically worsen neuroinflammation. ApoD directly counteracts neuronal ferroptosis by sustaining GSH levels and inhibiting lipid peroxidation. The elevated ApoD levels observed in the CSF of PD patients may represent a compensatory protective response. ApoE isoforms (except E4) suppress ferroptosis by activating the PI3K/AKT pathway, thereby inhibiting ferritinophagy and pathological iron release. ApoJ (Clusterin) indirectly inhibits ferroptosis by binding *α*-syn oligomers, suppressing their spread and reducing associated oxidative stress. In contrast, the ApoE4 isoform has detrimental effects: its unstable lipoprotein particles exacerbate neuronal lipid peroxidation and ferroptosis, significantly correlating with increased PD risk (Dai et al., 2025).

Conversely, harmful pathways are also activated. Upregulation of NOX4 in hippocampal astrocytes during PD promotes neuroinflammation and mitochondrial damage by increasing myeloperoxidase (MPO) and osteopontin (OPN). This cascade induces ferroptosis through elevated oxidative stress and impairment of the mitochondrial electron transport chain (Boonpraman et al., 2023). Notably, astrocytes also exhibit protective intercellular communication mechanisms; for instance, ferritin released by astrocytes can be taken up by neighbori ng MES23.5 DA neurons in co-culture systems. This ferritin transfer significantly alleviates MPP^+^(the active neurotoxic metabolite of MPTP)-induced damage and inhibits ferroptosis in DA neurons (Zhang et al., 2022).

DJ-1, which is predominantly expressed in human astrocytes, exerts multiple neuroprotective effects by regulating oxidative stress, maintaining mitochondrial function, and modulating anti-inflammatory responses (Miyazaki and Asanuma, 2020). Accordingly, DJ-1 knockout has been shown to impair astrocyte-mediated protection against 6-hydroxydopamine toxicity (Lev et al., 2013). Furthermore, loss of DJ-1 in astrocytes leads to hyperactivation of inflammatory responses upon LPS stimulation, resulting in excessive production of nitric oxide (NO), COX-2, and IL-6 via a ROS-p38 MAPK pathway (Waak et al., 2009). This dysregulation may contribute to PD pathogenesis by promoting nitrative stress.

Beyond these well-characterized roles, emerging evidence positions DJ-1 as a critical guardian against ferroptosis, a function highly relevant to astrocyte-mediated neuroprotection. The molecular mechanism, elucidated in cancer cells but likely conserved in neural cells, involves DJ-1 sustaining the transsulfuration pathway (Cao et al., 2020). As detailed in Section 2.1, DJ-1 maintains the activity of SAHH, ensuring the production of homocysteine and subsequently cysteine for GSH synthesis. In astrocytes, this function may be crucial for maintaining their own robust antioxidant reservoir, enabling them to effectively buffer neuronal oxidative stress and neutralize lipid peroxides. Furthermore, by securing their metabolic resilience against ferroptosis, DJ-1-competent astrocytes can better provide trophic support, regulate extracellular glutamate, and mitigate neuroinflammatory cascades, all processes that indirectly influence neuronal susceptibility to ferroptotic death (Cao et al., 2020).

Conversely, DJ-1 overexpression preserves astrocyte function and prevents oxidative stress. Astrocytic DJ-1 overexpression provides neuroprotection against MPP^+^-induced DA neuron loss and motor deficits. Proteomic analyses indicate that DJ-1 activates the Nrf2 pathway, modulating antioxidant responses and metabolic enzymes to counteract oxidative stress in transgenic zebrafish (Frøyset et al., 2018). This Nrf2 activation may work in concert with the SAHH-dependent maintenance of the transsulfuration pathway, creating a multi-layered defense system wherein DJ-1 coordinately enhances both the synthesis (via transsulfuration) and the utilization (via Nrf2-target genes like GCLM) of glutathione. These findings collectively highlight the multifaceted neuroprotective influence of astrocytic DJ-1 on DA neurons, extending from classical anti-inflammatory and antioxidant support to include the preservation of a key metabolic pathway that directly counters the execution of ferroptosis.

Astrocytes play a dual role in PD, acting both as protectors and contributors to neurodegeneration through mechanisms involving ferroptosis, oxidative stress, and neuroinflammation. Their ability to transition between neurotoxic and neuroprotective states highlights their dynamic influence on disease progression. Key pathways such as mTOR, Nrf2, and DJ-1 signaling offer promising targets for therapeutic intervention. Enhancing astrocyte-mediated defense mechanisms while suppressing harmful responses could pave the way for novel strategies to halt or slow neuronal loss in PD. Understanding the complex roles of astrocytes provides not only deeper insights into PD pathogenesis but also opportunities for developing astrocyte-centered therapies.

#### 5.2.3 Involvement of other cellular entities

Beyond microglia and astrocytes, the pathogenic milieu of PD involves several other cellular players. Oligodendrocytes, the myelinating cells of the CNS, can release excess iron under pathological conditions such as demyelination, thereby contributing to neuronal iron overload and ferroptosis. Notably, alterations in oligodendrocyte gene expression emerge early in PD, even before overt dopaminergic degeneration, pointing to their role in initial disease mechanisms. While dysfunctional oligodendrocytes may exacerbate neurodegeneration, they also exert protective effects, such as secreting the iron-storage protein FTH1 to counter iron-mediated cytotoxicity (Li M. et al., 2024). In addition, peripheral immune cells, including T-cells and monocytes, infiltrate the CNS in PD and modulate neuroinflammation. Their crosstalk with resident glia amplifies inflammatory responses and influences disease progression (Brodacki et al., 2008; Brochard et al., 2009). The blood–brain barrier (BBB) endothelial cells also play a critical role: they regulate the bidirectional transport of pathological proteins like *α*-synuclein, and their dysfunction impairs protein clearance while promoting aggregation, thereby accelerating PD pathology. Moreover, activated BBB endothelial cells exacerbate neuroinflammation by releasing cytokines and facilitating leukocyte infiltration into the brain, further amplifying neurodegeneration (Yuan et al., 2023). Thus, a comprehensive understanding of PD pathogenesis requires integrating this multicellular network, in which neuronal vulnerability is shaped by dysregulated interactions among neurons, glia, vascular cells, and peripheral immune components.

## 6 Therapeutic strategies targeting pathophenotypes

Emerging therapeutic strategies increasingly target the *oxytosis*/*ferroptosis* pathway, a promising axis for disease modification in neurodegenerative diseases including PD. A recent editorial underscores that ferroptosis, driven by oxidative stress, mitochondrial dysfunction, and immune dysregulation, is a key mechanistic node in PD and Alzheimer’s disease (AD), highlighting its potential as both a biomarker and a therapeutic target (Dar et al., 2024).

Among the compounds actively being explored, the flavonoid fisetin has demonstrated notable neuroprotection in PD preclinical models. Oral administration in neurotoxin MPTP (1-methyl-4-phenyl-1,2,3,6-tetrahydropyridine) and rotenone induced rodent models dose-dependently improved motor function, preserved striatal dopamine and tyrosine hydroxylase levels, and reduced DA neuron loss (Maher, 2021; Maher, 2017). Its efficacy is attributed to multi-target actions, including inhibiting ferroptosis via glutathione maintenance and reduced lipid peroxidation, as well as attenuating oxidative stress and neuroinflammation, and improving mitochondrial function, aligning well with PD’s complex pathophysiology.

Further expanding the therapeutic landscape, derivatives developed from a screening platform focused on ferroptosis have shown translational promise. CMS121, a fisetin derivative, partially inhibits fatty acid synthase (FASN), thereby reducing peroxidation-prone polyunsaturated fatty acids and subsequent lipid peroxidation. J147, a curcumin-derived compound, targets mitochondrial ATP synthase (ATP5A). Despite distinct primary targets, both converge on inhibiting acetyl-CoA carboxylase (ACC1), elevating acetyl-CoA levels, preserving mitochondrial homeostasis, and ultimately blocking ferroptotic cell death. While most extensively studied in AD models, their shared mechanism supports their potential relevance in PD and other age-related neurodegenerative conditions, including counteracting glutathione depletion, lipid peroxidation, and metabolic dysfunction. CMS121 has completed IND-enabling studies, and J147 has entered Phase 1 clinical trials, underscoring their pathway-driven therapeutic promise (Maher et al., 2020; Ates et al., 2020; Currais et al., 2019).

Building on specific therapeutic directions, the following sections will examine how neuronal and glial subtypes differentially contribute to ferroptotic vulnerability and propagation in the PD brain.

### 6.1 Targeting the synergy between *α*-synuclein aggregation and ferroptosis

Iron overload accelerates the formation of toxic α-syn oligomers, while aberrant α-syn expression disrupts iron metabolism and GSH synthesis, thereby creating a vicious cycle that enhances neuronal susceptibility to ferroptosis (Chen et al., 2025). Inhibiting the aggregation of α-syn or removing excess iron may represent potential therapeutic strategies for PD. This section examines therapeutic approaches aimed at mitigating α-syn-induced ferroptotic pathways in PD.

#### 6.1.1 α-synuclein-directed therapeutics

Several therapeutic strategies are currently focused on directly inhibiting the aggregation of α-syn. Among these, small molecules and antibodies designed to target the P1–P2 region of α-syn oligomers or fibrils aim to selectively suppress pathological aggregation. For instance, αSP1, a synthetic antibody (sybody) binding the P1 region (residues 36–42) of α-syn, effectively inhibits amyloid aggregation at low stoichiometries by engaging both oligomeric and fibrillar species. It disrupts primary and secondary nucleation processes, reduces cytotoxicity, and maintains specificity within complex biological environments, highlighting its potential as a therapeutic strategy for synucleinopathies such as PD (Gialama et al., 2024). In contrast, prasinezumab, a monoclonal antibody targeting aggregated *α*-syn, was evaluated in the phase 2 PASADENA trial (NCT03100149). Despite doses of 1,500 mg or 4,500 mg, no significant differences were observed compared to placebo in reducing MDS-UPDRS total scores (Parts I–III) at 52 weeks. Furthermore, the treatment showed no meaningful effects on dopamine transporter imaging or clinical progression, although infusion-related reactions were reported in 19–34% of participants. These results suggest a lack of disease-modifying effects in early-stage PD patients (Pagano et al., 2022). An ongoing phase 2b trial, PADOVA (NCT04777331), is now evaluating prasinezumab in an older cohort with longer disease duration and more severe motor symptoms. Outcomes are currently pending (Nikolcheva et al., 2025).

Another approach involves structure-based inhibitors that prevent *α*-syn misfolding and oligomerization, thereby reducing susceptibility to ferroptosis. The peptide inhibitor 4,554 W, derived from the preNAC region (residues 45–54) of α-syn and stabilized by N-acetylation and C-amidation, incorporates into *β*-sheet structures and interferes with fibril nucleation. In transgenic models, it reduced fibril formation by 62% and striatal deposits by 41%, while also improving motor function without eliciting significant immunogenicity. However, its clinical translation is hampered by poor blood–brain barrier penetration (Meade et al., 2020). Beyond peptide-based agents, small molecules such as SynuClean-D (SC-D) exhibit broad efficacy against various α-syn amyloid strains. SC-D inhibits aggregation, prevents seeded polymerization, and disaggregates preformed fibrils. It also reduces intracellular accumulation of phosphorylated α-syn inclusions in a strain-dependent manner. Although SC-D currently lacks optimal drug-like properties, it represents a valuable tool for proof-of-concept studies and high-throughput screening (Peña-Díaz et al., 2022).

Active immunotherapy has also been explored, as demonstrated by PD01A, which showed favorable tolerability in early PD patients over six immunizations administered across five years. Robust antibody responses with geometric mean titers reaching 1:20,218 confirmed target engagement. Booster immunizations effectively reactivated humoral immunity, supporting progression to phase 2 trials (Volc et al., 2020). Collectively, these findings reflect sustained interest in immunotherapeutic and aggregation-inhibiting strategies, despite mixed outcomes in clinical trials to date.

#### 6.1.2 Ferroptosis-α-synuclein intersection modulators

Emerging evidence highlights the interplay between α-syn aggregation and ferroptosis in PD, revealing multi-target therapeutic opportunities. Pyridoxal phosphate (PLP), the active coenzyme form of vitamin B6, activates GOT1 to enhance the methionine salvage pathway and increase GSH synthesis. This action suppresses α-synuclein-induced ferroptosis and protects DA neurons, highlighting its potential for repurposing in PD therapy (Min et al., 2025). Similarly, melatonin receptor MT1 deficiency exacerbates α-syn pathology and ferroptosis in PD models by disrupting the Sirt1/Nrf2/HO-1/GPX4 axis and promoting iron accumulation, whereas MT1 overexpression attenuates these effects (Lv et al., 2024). Natural products also demonstrate promise: the fungal-derived meroterpenoid granulathiazole A (GA) activates Nrf2/HO-1 signaling, inhibits ferroptosis via SLC7A11/GPX4/FSP1 upregulation and ACSL4 downregulation, and reduces α-syn accumulation across cellular and zebrafish models (Li et al., 2024a). Likewise, acteoside, a plant-derived compound, counteracts neuronal damage through PI3K/Akt-mediated Nrf2 activation, restoring redox balance and inhibiting ferroptosis (Wang et al., 2025). Together, these findings underscore the therapeutic potential of simultaneously targeting α-syn aggregation and ferroptosis pathways.

#### 6.1.3 Iron chelators

Deferiprone (DFP), an FDA-approved oral iron chelator capable of crossing the blood–brain barrier, reduces nigral iron content, suppresses α-syn aggregation, and protects DA neurons through dual inhibition of iron-dependent α-syn fibrillation and ferroptosis. In MPTP-induced PD mice, DFP significantly reduced iron deposition in the substantia nigra, increased DA neuron survival, and improved motor function (Zeng et al., 2021). However, clinical outcomes have been mixed: a phase II randomized controlled trial (RCT) in early-onset PD patients showed that short-term DFP treatment was safe but only trended toward motor improvement (Martin-Bastida et al., 2017). Another RCT in newly diagnosed PD patients found that DFP accelerated disease progression and necessitated earlier dopaminergic therapy (Devos et al., 2022). These trials indicate that DFP has not achieved desired efficacy and is associated with significant adverse effects, limiting its application in PD.

Dexrazoxane (Dex), a highly hydrophilic iron chelator, binds free iron and enters the brain through a compromised BBB. In PD animal models, intraperitoneal administration of Dex improved contralateral rotation behavior and motor dysfunction by reducing oxidative damage and systemic inflammation (Mei et al., 2019). Similarly, BJP-IVb protects against PD by suppressing iron overload-mediated ferroptosis of DA neurons. It inhibits IRP2, reduces iron transport and lipid peroxidation, and improves motor function (Li et al., 2024b). Although iron chelation remains conceptually appealing, clinical translation requires more targeted and tolerable agents.

The interplay between *α*-syn aggregation and ferroptosis forms a key pathogenic loop in PD. These therapeutic strategies offer promising avenues to disrupt this cycle. Although several agents have demonstrated compelling efficacy in preclinical studies, clinical outcomes for many candidates remain limited. Future research should prioritize combination therapies, enhance blood–brain barrier penetration, and develop patient stratification strategies based on iron deposition and α-syn pathology. A summary of these therapeutic approaches is provided in Table 2.

**Table 2:** Therapeutic strategies targeting the synergy between α-synuclein aggregation and ferroptosis.

Therapeutic strategy category	Specific approach/agent	Primary mechanism of action	Current status/clinical outcomes	References
α-synuclein-Directed Therapeutics	αSP1	a synthetic antibody binding the P1 region of α-syn	Inhibited amyloid aggregation and disrupted primary and secondary nucleation processes	Gialama et al. (2024)
	Prasinezumab	Targets aggregated α-syn	PASADENA showed no significant clinical improvement in early PD. PADOVA is ongoing.	Pagano et al. (2022) and Nikolcheva et al. (2025)
	4,554 W	Incorporates into β-sheet structures.	Reduced fibrils and deposits in transgenic models; Limited by poor BBB penetration.	Meade et al. (2020)
	SC-D	↓ aggregation, prevents seeded polymerization, and disaggregates fibrils.	Preclinical efficacy against various strains. Lacks drug-like properties; useful for research and screening.	Peña-Díaz et al. (2022)
	PD01A	↓ antibody responses targeting α-syn.	Good tolerability and robust antibody titers in early PD patients.	Volc et al. (2020)
Ferroptosis-α-syn Intersection Modulators	PLP	Activates GOT1 to ↑ GSH	Preclinical evidence of neuroprotection. Clinical studies warranted.	Min et al. (2025)
	MT1 Modulation	Activates Sirt1/Nrf2/HO-1/GPX4 pathway	Preclinical evidence	Lv et al. (2024)
	GA	Activates Nrf2/HO-1, upregulates SLC7A11/GPX4/FSP-1	Effective in cellular and zebrafish PD models.	Li et al. (2024a)
	Acteoside	Activates Nrf2 via PI3K/Akt, restoring SLC7A11/GPX4	Counters neurotoxicity in PD models.	Wang et al. (2025)
Iron Chelators	DFP	Oral chelator that reduces nigral iron	Preclinically effective. Clinical trials showed safety but mixed/motor results	Zeng et al. (2021), Martin-Bastida et al. (2017), and Devos et al. (2022)
	Dex	Binds free iron, ↓oxidative damage	Improved motor function in PD animal models.	Mei et al. (2019)

↑: up-regulate or increase; ↓: down-regulate or decrease.

### 6.2 Modulating glial amplification loops

#### 6.2.1 Therapeutic strategies targeting microglial ferroptosis

##### 6.2.1.1 Microglial polarization modulation and ferroptosis inhibition

Microglia play a crucial role in the pathogenesis of PD, particularly through promoting ferroptosis, a process exacerbated by pro-inflammatory M1 polarization. This activation enhances iron accumulation and lipid peroxidation, accelerating neuronal damage. Modulating microglial polarization toward the anti-inflammatory M2 phenotype has thus emerged as a promising therapeutic strategy to suppress neuroinflammation and ferroptosis, thereby slowing disease progression.

For instance, Z-ligustilide has been shown to activate the Nrf2-TrxR axis, shifting microglia toward the protective M2 phenotype. This transition attenuates oxidative stress and neuroinflammation, ultimately preserving DA neurons (Peng et al., 2024). Similarly, CPT improved motor function and reduced neuronal loss in LPS-induced PD models by balancing microglial polarization via the AKT/Nrf2/HO-1 and NF-κB pathways, further underscoring the importance of microglial phenotype regulation in neuroprotection (He et al., 2021). Additionally, ceftriaxone (CEF) inhibits microglial activation and reduces neuroinflammation by suppressing ferroptosis through upregulation of SLC7A11 and GPX4, thereby protecting DA neurons in PD models (Zhi et al., 2025). In summary, these studies highlight microglial polarization modulation as a central therapeutic approach in PD, targeting both inflammatory response and iron-dependent oxidative damage to confer neuroprotection.

##### 6.2.1.2 Nanoformulations for targeted microglial modulation

Although conventional interventions targeting microglia can alleviate neuroinflammation in the short term, immune disorders mediated by peripheral inflammatory cells lead to continuous infiltration and contribute to the overactivation of the immune microenvironment in PD. To address this, extracellular vesicle-based nanoformulations (EVNs) have been developed, which consist of CCR2-enriched mesenchymal stem cell-derived extracellular vesicles as the shell and a dihydrotanshinone I (DT)-loaded diselenide-bridged mesoporous silica nanoparticle as the core. This strategy combines “internal” anti-inflammatory effects with “external” immune regulation (Zhang C. et al., 2024). Specifically, EVNs target and block the infiltration of peripheral inflammatory cells via the CCR2–CCL2 axis, while the released DT activates the Nrf2–GPX4 pathway, inhibiting microglial ferroptosis and neuroinflammation, thereby promoting a shift toward an anti-inflammatory phenotype.

In another approach, a nanomedicine termed QAE NPs was designed based on stem cell-derived exosomes co-loaded with hydroxyl-terminated phosphorus dendrimers (AK76) and quercetin (Que). This system enables nose-to-brain delivery without crossing the blood–brain barrier and exhibits synergistic anti-inflammatory, antioxidant, and neuroprotective effects. It effectively ameliorates motor deficits and depressive-like behaviors in PD mice by simultaneously modulating microglia and neurons (Zhang et al., 2025d). Furthermore, biomimetic nanoparticles have been shown to enhance the recovery of motor functions in PD by improving microglial mitochondrial homeostasis and suppressing neuroinflammation (Li et al., 2025).

##### 6.2.1.3 LRRK2 kinase inhibitors: from mechanism to clinical translation

As an important direction in potential treatment strategies for PD, inhibitors targeting the LRRK2 kinase have attracted significant attention in recent years. By inhibiting LRRK2 activity and modulating downstream lysosomal function, these compounds hold promise for slowing disease progression. Among them, DNL201, a CNS-penetrant LRRK2 inhibitor, has been shown in preclinical studies to effectively reduce LRRK2 activity and restore lysosomal function. Subsequent clinical trials in healthy volunteers and patients with PD further confirmed its target engagement, biomarker modulation, favorable safety profile, and tolerability, providing strong support for the advancement of LRRK2 inhibitors for the treatment of PD (Jennings et al., 2022). Another study evaluated BIIB122 (DNL151), a potent and selective LRRK2 inhibitor, which demonstrated dose-dependent LRRK2 inhibition and modulation of the lysosomal pathway in both healthy participants and PD patients. This compound also exhibits good central nervous system penetration and a favorable safety profile, supporting its further development as a therapeutic agent for PD (Jennings et al., 2023). Collectively, these studies lay a solid foundation for the clinical application of LRRK2 inhibitors in the treatment of PD.

##### 6.2.1.4 Non-pharmacological interventions

Although microglia may express the xCT system, its expression level, functional significance, and impact on overall brain antioxidant status are considerably less pronounced than those in astrocytes. Its role appears to be more closely associated with redox regulation under inflammatory conditions. Interestingly, non-pharmacological interventions such as physical exercise (PE) demonstrate protective effects by targeting this pathway in microglia. Studies in PD mouse models have shown that PE upregulates the cystine transporter SLC7A11, inhibits the pro-ferroptotic lipid oxygenase ALOX12, enhances microglial phagocytosis of *α*-syn, reduces markers of microglial ferroptosis, and ameliorates neurofunctional deficits. These findings suggest that microglia mediate, at least partially through the SLC7A11–ALOX12 axis, the beneficial effects of exercise in suppressing ferroptosis (Xu J. et al., 2025). In summary, microglial xCT activity may play a context-dependent role in neuroprotection, particularly under inflammatory stimulation, and physical exercise emerges as a promising non-pharmacological strategy to modulate microglial redox homeostasis and ferroptosis, thereby contributing to functional recovery in PD.

In summary, current therapeutic strategies for PD increasingly focus on modulating microglial function through diverse mechanisms, including polarization shift, ferroptosis inhibition, nano-enabled targeting, and kinase modulation. Both pharmacological agents and innovative nanoformulations show promise in mitigating neuroinflammation and oxidative damage, while non-pharmacological approaches like physical exercise offer complementary benefits through redox pathway regulation. The continued development of LRRK2 inhibitors and biomimetic nanotherapies highlights the translational potential of mechanism-based interventions. Together, these advances reflect a growing emphasis on precision targeting of glial pathways and integrated neuro-immune modulation, paving the way for more effective and multifaceted treatment paradigms in PD. A summary of these therapeutic approaches is provided in Table 3.

**Table 3:** Therapeutic strategies targeting microglial ferroptosis.

Therapeutic strategy category	Specific approach/agent	Primary mechanism of action	Current status/clinical outcomes	References
Microglial Polarization Modulation	Z-ligustilide	Activates Nrf2-TrxR axis to shift microglia to M2 phenotype.	Preclinically effective; attenuates neuroinflammation and preserves neurons.	Peng et al. (2024)
	CPT	Balances microglial polarization via AKT/Nrf2/HO-1 and NF-κB pathways.	Improved motor function and reduced neuronal loss in models.	He et al. (2021)
	CEF	↓ microglial activation and ↓neuroinflammation.	Demonstrated neuroprotective effects in PD models.	Zhi et al. (2025)
Nanoformulations for Microglia	EVNs	Block peripheral cell infiltration; activate Nrf2-GPX4 pathway.	Preclinical strategy combining external immune regulation and internal anti-inflammatory effects.	Zhang C. et al. (2024)
	QAE NPs	Enables nose-to-brain delivery for synergistic anti-inflammatory, antioxidant, and neuroprotective effects.	Ameliorates motor and depressive-like behaviors in PD mice.	Zhang et al. (2025d)
	Biomimetic Nanoparticle	↑ microglial mitochondrial homeostasis and ↓neuroinflammation.	Enhances recovery of motor functions in PD models.	Li et al. (2025)
LRRK2 Kinase Inhibitors	DNL201	CNS-penetrant inhibitor; ↓ LRRK2 activity and restores lysosomal function.	CNS-penetrant inhibitor; reduces LRRK2 activity and restores lysosomal function.	Jennings et al. (2022)
	DNL151	Potent and selective LRRK2 inhibitor; modulates lysosomal pathway.	Shows dose-dependent inhibition, good CNS penetration, and favorable safety in healthy participants and PD patients.	Jennings et al. (2023)
Non-Pharmacological Intervention	Physical Exercise	↑ microglial SLC7A11, ↓ALOX12, ↑α-syn phagocytosis.	Ameliorates neurofunctional deficits in PD mouse models.	Xu J. et al. (2025)

↑: up-regulate or increase; ↓: down-regulate or decrease.

#### 6.2.2 Therapeutic strategies targeting astrocytic ferroptosis

Astrocytes, the most abundant glial cells in the central nervous system, play essential roles in supporting neuronal function, regulating antioxidant defense, and maintaining metabolic homeostasis.

Activation of the Nrf2 signaling pathway in astrocytes provides neuroprotection by promoting the production of various antioxidant molecules. This pathway serves as a critical regulator of cellular redox homeostasis, particularly under oxidative stress conditions such as those found in PD. Targeting astrocytic serotonin 1A (5-HT1A) receptors with (R)-(+)-8-hydroxy-2-(di-n-propylamino)tetralin hydrobromide (8-OH-DPAT) stimulates astrocyte proliferation and upregulates metallothionein (MT-1/−2) expression via Nrf2 nuclear translocation. This process reduces DA neuron loss and enhances astrocytic metallothionein levels (Miyazaki et al., 2013).

Additionally, the anti-Parkinson’s drug rotigotine increases striatal astrocytic MT-1/2 and protects nigral DA neurons in a 6-hydroxydopamine (6-OHDA) mouse model of PD through activation of astrocytic 5-HT1A receptors, highlighting its potential as a neuroprotective therapy against dopaminergic degeneration (Isooka et al., 2020). Beyond receptor-specific agents, other agents also exert neuroprotective effects by activating the Nrf2 pathway. For instance, the probiotic strain *Lactococcus lactis* MG1363-pMG36e-GLP-1 activates the Nrf2/GPX4 signaling pathway to inhibit ferroptosis, ameliorating motor deficits in PD mice (Yue et al., 2022). Moreover, therapeutic compounds such as melatonin, salidroside, and quercetin demonstrate neuroprotective properties in PD models via activation of the Nrf2/GPX4 pathway (Lv et al., 2024; Shen J. et al., 2024; Lin et al., 2022). These findings collectively underscore the broad therapeutic potential of targeting the Nrf2 system within astrocytes to mitigate oxidative damage and ferroptosis in PD.

The system Xc^−^–GPX4 axis represents a central mechanism in the cellular defense against ferroptosis, serving as a critical regulatory node in maintaining redox balance and lipid peroxidation homeostasis. Studies have shown that the antiepileptic drugs levetiracetam (LEV) and zonisamide (ZNS) protect DA neurons from 6-OHDA-induced toxicity by upregulating the xCT transporter in astrocytes, thereby increasing GSH levels (Miyazaki et al., 2016; Asanuma et al., 2010). This underscores the broader relevance of repurposing existing drugs for neuroprotection through astrocyte-mediated mechanisms. Furthermore, activation of the system Xc^−^–GPX4 pathway enhances antioxidant capacity and represents a promising therapeutic strategy. In an MPTP-induced mouse model of PD, oral administration of total flavonoids of Astragalus (TFA) increased GPX4 expression, elevated GSH levels, reduced ROS production, and suppressed neuronal ferroptosis (Gao et al., 2024). Subsequent clinical double-blind controlled trials indicated that multi-dose flavonoid regimens (200–800 mg daily) were safe, well-tolerated, and without significant accumulation, supporting the potential of flavonoid-based supplements in PD management (Pang et al., 2016). These results bridge preclinical findings with clinical applicability, highlighting flavonoids as feasible adjunct therapeutics. GSH, a critical antioxidant, plays a vital role in neuronal protection. N-Acetylcysteine (NAC), a precursor of GSH, helps sustain redox homeostasis and mitigates oxidative damage. Intravenous NAC administration significantly increases dopamine transporter (DAT) binding in the caudate and putamen, correlating with marked clinical improvement (Monti et al., 2019). These findings underscore that astrocytic xCT-GPX4 represents a key target for neuroprotection, offering a novel therapeutic strategy for preventing dopaminergic neurodegeneration.

Astrocytes significantly contribute to the regulation of ferroptosis in PD through mechanisms involving the Nrf2 pathway and system Xc^−^–GPX4 signaling. Therapeutic targeting of astrocytic receptors and antioxidant pathways presents a promising direction for developing neuroprotective treatments aimed at preserving DA neurons and slowing disease progression.

## 7 Conclusion

The pathogenesis of PD is driven by a synergistic, self-reinforcing cycle between *α*-syn pathology and ferroptosis that collectively accelerates neurodegeneration. This vicious cycle is critically amplified by the involvement of non-neuronal cells, primarily microglia and astrocytes. Microglia, which exhibit intrinsic vulnerability to ferroptosis, adopt a pro-inflammatory phenotype under iron overload, releasing cytotoxic cytokines and reactive oxygen species that exacerbate neuronal damage. Astrocytes, in contrast, play a dual role: while their dysfunction can promote oxidative stress and calcium dysregulation, they also retain protective mechanisms such as the Nrf2-GPX4 antioxidant pathway, representing a potential therapeutic target. The early appearance of non-motor symptoms, years before overt motor signs emerge, implies that early glial involvement, particularly microglia-driven ferroptosis, may serve as a critical initiating event. This underscores the imperative for early intervention and deeper mechanistic investigation into microglial activation to develop strategies that halt disease progression at its initial stages.

Guided by this pathophysiology, therapeutic efforts are increasingly aimed at disrupting this multifaceted cascade. Current strategies include directly targeting α-syn aggregation via immunotherapies and small molecules, employing iron chelators to mitigate overload, and reinforcing endogenous antioxidant defenses. Furthermore, modulating glial activity—by steering microglia toward an anti-inflammatory phenotype or enhancing astrocytic protective functions—constitutes a promising approach to counteract neuroinflammation and oxidative stress. The emergence of novel nanoformulations and drug repurposing initiatives continues to expand the therapeutic landscape.

Although prior reviews have established the fundamental involvement of ferroptosis in PD, our work extends the existing literature through several distinct contributions (Ding X. S. et al., 2023). First, we accentuate the bidirectional vicious cycle between *α*-syn aggregation and ferroptosis, elaborating not only on how α-syn disrupts iron homeostasis and drives lipid peroxidation, but also how iron and its associated oxidative metabolites directly stabilize α-syn oligomers and accelerate fibrillation. Second, we provide a systematic and updated overview of glial contributions, highlighting emerging evidence that microglia act as primary drivers of iron-mediated neurotoxicity, and that astrocytes undergo dynamic phenotypic shifts regulated by pathways such as mTOR and CSE/YAP/FOXD3 mechanisms that modulate neuronal vulnerability in previously underappreciated ways. Third, we compile emerging therapeutic strategies not comprehensively addressed in earlier reviews, including structure-based α-syn aggregation inhibitors (e.g., αSP1, 4,554 W), glia-targeting nanoformulations, LRRK2 kinase inhibitors in clinical translation, and non-pharmacological interventions such as physical exercise—all contextualized within the α-syn–ferroptosis axis.

In our view, the complexity of PD mandates a combinatorial therapeutic paradigm. Future success will likely hinge on multi-target strategies that simultaneously inhibit α-syn toxicity, rectify iron dyshomeostasis, and harness the supportive functions of glia—all tailored to disease stage and individual patient profiles. Targeting the intersection of α-syn and ferroptosis provides a compelling framework for developing the next generation of disease-modifying therapies. This integrated perspective not only refines our mechanistic understanding but also unveils innovative avenues for effective intervention in PD.

### 7.1 Future perspectives

Despite significant progress in linking ferroptosis to PD, several key challenges and opportunities remain. First, there is a need to develop more sensitive and specific biomarkers for detecting ferroptosis *in vivo* in the human brain. Advanced imaging techniques combined with biochemical assays for lipid peroxidation products (e.g., specific isoprostanes) and iron speciation in cerebrospinal fluid or blood could enable earlier diagnosis and improved patient stratification. Second, the temporal and spatial dynamics of glial–iron–*α*-syn interactions require further elucidation. Longitudinal studies in animal models and post-mortem human tissues are needed to determine whether microglial iron overload is a cause or consequence of α-syn pathology, and to identify the disease stage at which glial modulation is most therapeutically beneficial. Third, translating preclinical findings into clinical success remains a major hurdle. Future drug development should prioritize compounds with excellent blood–brain barrier penetration and favorable safety profiles. Combination therapies targeting multiple nodes of the α-syn–ferroptosis–glial axis (e.g., an iron chelator plus an α-syn aggregation inhibitor) may yield synergistic effects and overcome compensatory resistance. Fourth, the roles of other non-neuronal cells, including oligodendrocytes, peripheral immune cells, and gut–brain axis components, in modulating ferroptosis warrant deeper investigation. Finally, personalized medicine approaches should be explored, taking into account genetic backgrounds (e.g., LRRK2 and SNCA mutations) and environmental exposures that influence iron metabolism and susceptibility to ferroptosis. Addressing these questions will not only refine our understanding of PD pathogenesis but also accelerate the development of effective, disease-modifying therapies.

## Glossary

4-HNE: 4-Hydroxynonenal
6-OHDA: 6-Hydroxydopamine
ACC1: Acetyl-CoA Carboxylase 1
ACE2: Angiotensin-Converting Enzyme 2
ACSL4: Acyl-CoA Synthetase Long Chain Family Member 4
AD: Alzheimer’s Disease
AHCYL1: S-adenosylhomocysteine hydrolase-like 1 protein
AK76: Hydroxyl-Terminated Phosphorus Dendrimers
ALOX15: Arachidonate 15-Lipoxygenase
ALP: Autophagy-Lysosome Pathways
AREs: Antioxidant Response Elements
ATP5A: ATP Synthase Subunit alpha
BBB: Blood–Brain Barrier
BH4: Tetrahydrobiopterin
CEF: Ceftriaxon
CNMT: Carnosine-N-Methyltransferase
CNS: Central Nervous System
CoQ10: Coenzyme Q10
CP: Ceruloplasmin
CSE: Cystathionine *γ*-lyase
CSF: Cerebrospinal Fluid
DA: Dopaminergic
DAT: Dopamine Transporter
Dex: Dexrazoxane
DFP: Deferiprone
DHODH: Dihydroorotate Dehydrogenase
DJ-1: Parkinsonism-associated Deglycase.
DLB: Dementia with Lewy Bodies
DT: Dihydrotanshinone I
ether-PL: ether-Phospholipid
EVNs: Extracellular Vesicle-Based Nanoformulations
FASN: Fatty Acid Synthase
FGF: Fibroblast Growth Factor
FOXD3: Forkhead Box D3
FPN: Ferroportin
FSP1: Ferroptosis Suppressor Protein 1
FTH1: Ferritin Heavy Chain 1
GA: Granulathiazole A
GCH1: GTP Cyclohydrolase 1
GPX4: Glutathione Peroxidase 4
GR: Glutathione Reductase
GS: Glutathione Synthetase
GSH: Glutathione
GSSG: Oxidized Glutathione
GWAS: Genome-Wide Association Studies
HMOX1: Heme Oxygenase 1
HO-1: Heme Oxygenase 1
HSPB1-Heat Shock Protein Beta-1
IFN-γ: Interferon-gamma
IPMDS: International Parkinson and Movement Disorders Society
IRE: Iron-Responsive Element
IRP1: Iron Regulatory Protein 1
IRP2: Iron Regulatory Protein 2
LBs: Lewy Bodies
LEV: Levetiracetam
LOX: Lipoxygenase
LPCAT3: Lysophosphatidylcholine Acyltransferase 3
LPS: Lipopolysaccharide
LRP1: Low-density Lipoprotein Receptor-related Protein 1
LRRK2: Leucine-Rich Repeat Kinase 2
MAO: Monoamine Oxidase
MDA: Malondialdehyde
MPO: Myeloperoxidase
MPP^+^: 1-Methyl-4-phenylpyridinium
MPTP: 1-Methyl-4-phenyl-1,2,3,6-tetrahydropyridine
MSA: Multiple System Atrophy
MT1: Melatonin Receptor 1A
MTs: Metallothioneins
NAC: N-Acetylcysteine
NCOA4: Nuclear Receptor Coactivator 4
NO: Nitric Oxide
NOX2: NADPH Oxidase 2
Nrf2: Nuclear Factor Erythroid 2-Related Factor 2
ONE: 4-oxo-2-nonenal
OPG: Osteoprotegerin
OPN: Osteopontin
p53: Tumor Protein p53
PD: Parkinson’s Disease
PDD: Parkinson’s Disease Dementia
PE: Physical Exercise
PINK1: PTEN-induced putative kinase 1
PLP: Pyridoxal Phosphate
PL-PUFAs: Phospholipid-Polyunsaturated Fatty Acids
PQS: Pseudomonas Quinolone Signal
PRKN: Parkin RBR E3 Ubiquitin-Protein Ligase
PUFAs: Polyunsaturated Fatty Acids
PV-INs: Parvalbumin-Positive Interneurons
QSM: Quantitative Susceptibility Mapping
Que.: Quercetin
RCT: Randomized Controlled Trial
ROS: Reactive Oxygen Species
SAT1: Spermidine/Spermine N1-Acetyltransferase 1
SC-D: SynuClean-D
SAHH: S-Adenosylhomocysteine Hydrolase
SLC40A1: Solute Carrier Family 40 Member 1
SLC7A11: Solute Carrier Family 7 Member 11
SN: Substantia Nigra
SNCA: Alpha-Synuclein Gene
SNc: Substantia Nigra pars compacta
SWI: Susceptibility-Weighted Imaging
TAG: Triacylglycerol
Tf: Transferrin
TFA: Total Flavonoids of Astragalus
TfR: Transferrin Receptor
TNF-*α*: Tumor Necrosis Factor Alpha
UPS: Ubiquitin-Proteasome System
VPS35: Vacuolar Protein Sorting 35
YAP: Yes-Associated Protein
ZNS: Zonisamide
α-syn: α-synuclein
